# Postoperative excessive external femoral rotation in revision total hip arthroplasty is associated with muscle weakness in iliopsoas and gluteus medius and risk for hip dislocation

**DOI:** 10.1186/s13018-021-02744-4

**Published:** 2021-10-09

**Authors:** Hyonmin Choe, Naomi Kobayashi, Daigo Kobayashi, Shintaro Watanabe, Koki Abe, Taro Tezuka, Yusuke Kawabata, Masanobu Takeyama, Yutaka Inaba

**Affiliations:** 1 Department of Orthopaedic Surgery, Yokohama City University, 3-9 Fukuura, Kanazawa-ku, Yokohama City, Kanagawa, 236-0004 Japan; 2 Department of Orthopaedic Surgery, Yokohama City University Medical Center, 4-57 Urahune-cho, Minami-ku, Yokohama City, Kanagawa, 232-0024 Japan

**Keywords:** Revision total hip arthroplasty, Femoral rotation, Functional stem anteversion, Muscle atrophy, Impingement, Dislocation

## Abstract

**Background:**

Excessive external femoral rotation (FR) can functionally increase stem anteversion (SA) and is often observed at an early stage after surgery in revision total hip arthroplasty (THA). This study was conducted to investigate the prevalence of external FR, identify the factors associated with external FR, and determine the association of FR and other factors with hip dislocation in revision THA.

**Methods:**

We enrolled 51 revision THA patients (55 hip cases). The patient background, angle of anatomical and functional SA, FR angle, sizes and densities of muscles around the hip joint, impingement distance, and consequence of postoperative hip dislocation were assessed by reviewing their medical history and imaging data that includes computed tomography (CT) scans before and after surgery.

**Results:**

Forty-five hip cases (81.8%) showed external FR (mean 13.0°). External FR was significantly correlated with anatomical SA (*r* =  − 0.54) and increase in functional SA (*r* = 0.36), which was significantly correlated with impingement distance (*r* = 0.46). The independent factors associated with external FR in multivariate analysis were the anatomical SA, CT densities of the psoas, gluteus medius and maximus muscles, and 2-stage revision (*R*^2^ = 0.559). During follow-up period, eight cases of revision THA showed hip dislocation. FR, functional SA, impingement distance, CT density of psoas and gluteus medius muscle, body mass index, number of past operation, and ratio of 2-stage revision THA were significantly different between cases with dislocation and non-dislocation. The odds ratio of FR and impingement distance for hip dislocation was identified as 1.061(95% confidence interval (CI): 1.011–1.114) and 0.901 (95% CI 0.820–0.991), respectively.

**Conclusions:**

Revision THA frequently causes an external FR that functionally increases the SA and impingement risk, particularly in hips with 2-stage revision with psoas and gluteus medius muscle atrophy. Patients who have undergone revision THA and have an excessive external FR may require careful monitoring for possible hip dislocation due to hip joint instability and impingement.

## Background

Using the combined anteversion technique for the cup and stem anteversion (SA) angle can minimize the risk of hip impingement during daily hip motions in total hip arthroplasty (THA) or revision THA patients [1–4]. Recent studies demonstrated the important effects of pelvic and femoral positions on anatomical and functional changes in the cup and SA angles, revealing the importance of tailored planning for the cup and SA that depends on the posture of THA patients [5–7]. Recurrent dislocation is among the major complications of THA, and the incidence rate of dislocation is higher in revision THA patients than in primary THA patients [8, 9]. In revision cases, excessive external rotation of the femur is often observed at an early stage after surgery, although several studies demonstrated that internal changes occur in the femoral position of primary THA patients [4, 7]. An external femoral rotation (FR) in revision THA patients may increase the functional angle of SA or risk of prosthetic or bony impingement and risk of hip dislocation in leg extension position, but this relationship has not yet been investigated.

The muscles around the hip joint affect its stability, which is an important factor in preventing postoperative hip dislocation in THA patients [10–12]. The muscles may also affect the FR alignment. Hence, muscle atrophy due to surgical damage or disuse may have a greater effect on the postoperative femoral position in revision THA patients than in primary THA patients. Previous studies focused on the effect of abductor muscles on hip dislocation [10, 13–15]; however, the association of muscles around the hip joint (including the psoas, iliac, and gluteus maximus (Gmax) and medius (Gmed)) with the FR angle or hip dislocation remains unclear.

Therefore, this study was conducted to investigate the prevalence of external FR in revision THA patients, identify the factors associated with external FR, and determine the association of FR and the other factors with the risk of hip dislocation in revision THA.

## Methods

### Patient analysis

**Fig. 1:**
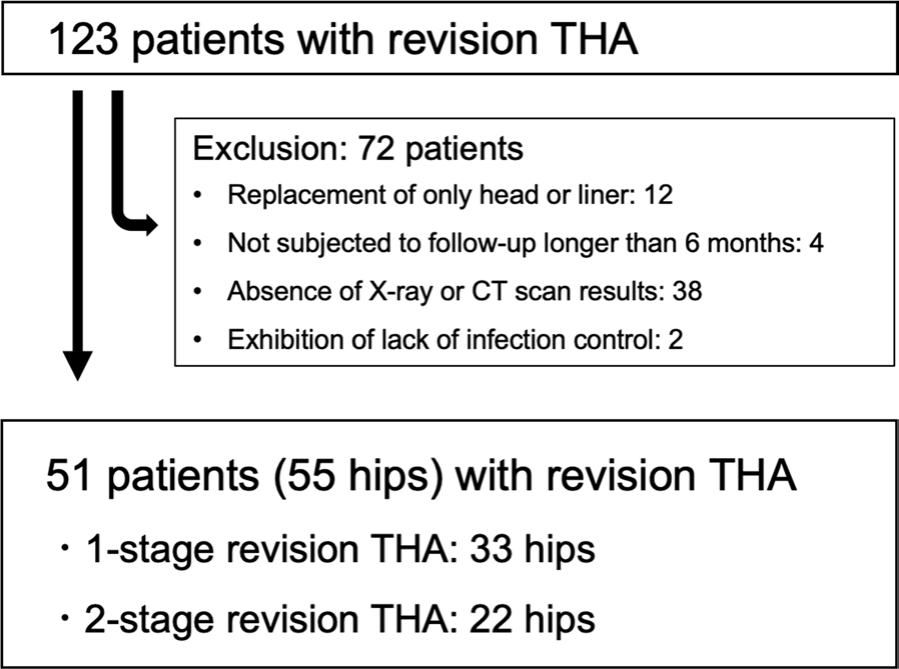
Flowchart of revision total hip arthroplasty (THA) patient enrolment

This retrospective study was approved by the institutional ethics review board of Yokohama City University (B200100008). The records of 123 patients who underwent revision THA in Yokohama City University Hospital from 2006 to 2019 were reviewed. The exclusion criteria were as follows: replacement of the head or liner only, uncontrollable periprosthetic joint infection (PJI), did not provide consent to participate, no follow-up after 6 months, and failure to undergo X-ray and computed tomography (CT) scans both before and after revision THA (Fig. 1). Our study cohort included 55 hip cases in 51 patients (37 women and 14 men) with a mean age of 70 years and mean body mass index (BMI) of 23.4 (kg/m^2^) (Table 1). All study participants underwent a hip X-ray in the frontal view and CT scan (Sensation 16; Siemens AG, Erlangen, Germany; 120 kV and 300 mA, 1.5 mm slice) from the first lumbar spine to the lower edge of femur before and within a month of surgery using a calibration phantom (B-MAS 200; Kyoto-Kagaku, Japan) and dedicated software (SYNAPSE Enterprise-PACS, Fujifilm, Tokyo, Japan). Muscle CT densities, measured in Hounsfield units, were adjusted based on the reference values from the calibration phantom [16].

**Table 1:** Demographic, surgical, and CT data in the revision THA cohort

Demographic data	
Age (years)	70.2 (9.6)
Female ratio (%)	73
Body mass index (kg/m^2^)	23.4 (4.8)
Diagnosis (hips)	Aseptic: 32, septic: 23
Preoperative femoral rotation	11.3 (19.1)
Surgical data	
Surgical procedure (hips)	1-stage: 33; 2-stage: 22
Leg length extension (mm)	11.9 (18.3)
Change of femoral offset (mm)	− 0.1 (9.5)
Postoperative anatomical stem anteversion	24.7 (14.7)
Preoperative muscle sizes (mm^2^)	
Psoas muscle	500 (31.8)
Iliac muscle	555 (39)
Gluteus maximus	1926 (660)
Gluteus medius	1688 (849)
Preoperative CT density of muscles	
Psoas muscle (HU)	35.2 (3.3)
Iliac muscle (HU)	55.3 (46.0)
Gluteus maximus (HU)	4.2 (2.9)
Gluteus medius (HU)	12.1 (35.3)

Data represent the mean values (standard deviation)

HU, Hounsfield unit

In the 55 revision THA cases, 23 hip cases were diagnosed with PJI using the modified Musculoskeletal Infection Society criteria [17] and 32 hip cases were diagnosed with aseptic loosening, including one pseudotumor. All 32 aseptic loosening patients underwent 1-stage revision surgery. Among the 23 PJI patients, one patient underwent 1-stage revision surgery and 22 patients underwent 2-stage THA using a hydroxyapatite block containing an antimicrobial (Bone Ceram P, Olympus Terumo Biomaterials Corp, Tokyo, Japan) (Table1) [18]. During the study period, 8 hips in 8 patients experienced postoperative dislocation, and all 8 patients were enrolled in the current study. Among these patients, 6 cases were conservatively treated and 2 cases were subjected for re-revision THA.

**Fig. 2:**
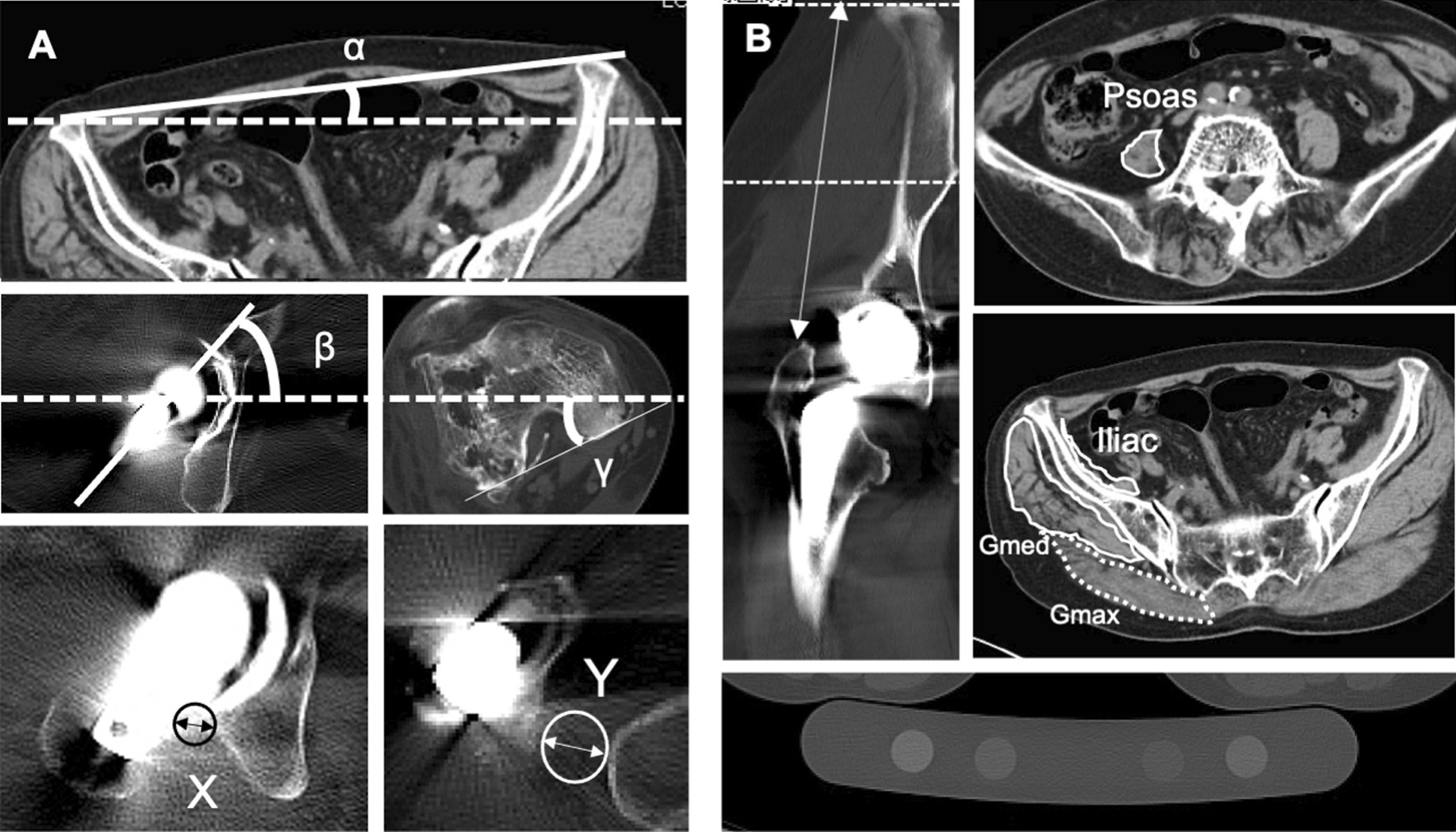
Computed tomography (CT) measurements of femoral rotation angle, impingement distance, and muscle size and density. **A** Functional stem anteversion or external rotation angle of the femur was measured by subtracting the value for the rotation angle of the superior anterior iliac spine line from that of the stem angle (β–α) or angle of the posterior femoral condyle axis (γ–α) using a CT image acquired within a month of surgery. The posterior pelvis–stem or pelvis–femur distances were measured by drawing a circle and by measuring the shortest distance from the stem to the pelvis (X) or from the femur to pelvis (Y) at the lower level of the artificial head in a cross-sectional CT image acquired after surgery. **B** Using preoperative CT images, the psoas muscle was detected at the upper edge of the iliac crest, and the iliac, gluteus medius, and gluteus maximus muscles were detected at the level of the mid-point between the iliac crest and great trochanter. A calibration phantom was placed during the CT scan and utilized to adjust the CT density, which was measured in Hounsfield units. (Gmed, gluteus medius; Gmax, gluteus maximus)

Patient characteristics were investigated. The leg and femoral offset extension levels before and after surgery were investigated by performing a frontal view hip X-ray. FR alignments were assessed by measuring the anatomical and functional SA angle and FR angle using cross-sectional CT imaging before and after surgery (Fig. 2A) [4, 7]. The shortest posterior pelvis–stem distance was measured as the prosthetic impingement distance, and the shortest posterior pelvis–femur distance was measured as the bony impingement distance (Fig. 2A) using cross-sectional CT imaging after surgery. The shortest distance between the pelvis–stem and pelvis–femur was defined as the impingement distance. As a representative measure of muscle quality, preoperative cross-sectional CT images were used to evaluate the sizes and densities of the psoas, iliac, Gmax, and Gmed muscles using the calibration phantom (Fig. 2B) [16]. The prevalence of external FR in revision THA patients, factors associated with external FR, and association of these factors with the risk of hip dislocation in revision THA were investigated.

### Statistical analysis

Statistical significance was determined using Student’s *t* test or nonparametric Mann–Whitney U test. Correlations were analyzed using Pearson’s correlation analysis. A forward–backward selection method was used for multiple regression analysis. The odds ratio for dislocation was calculated by logistic regression analysis. All statistical analyses were conducted using JMP® 15 software (SAS Institute, Inc., Cary, NC, USA). P values < 0.05 were considered to indicate statistically significant results. All graphs were prepared using GraphPad Prism 8 software (GraphPad, Inc., CA, USA).

## Results

**Fig. 3:**
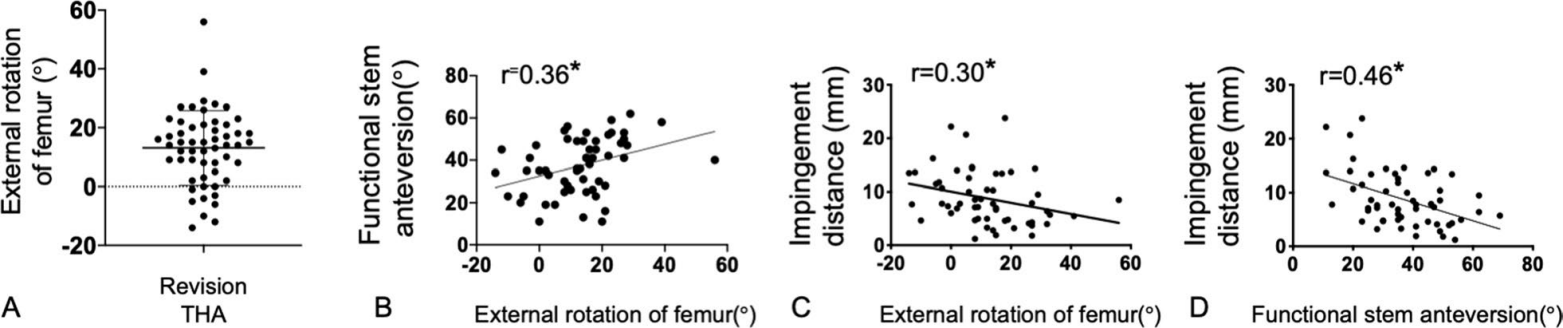
Femoral rotation (FR) angle and stem anteversion (SA) in revision and impingement distance. **A** External FR angle mean of 13.0° up to 56°. Among the 55 revision THA cases, 45 cases showed an external rotation, and 14 showed an external rotation of more than 30°. **B** External FR is correlated with functional SA in both revision and primary THA patients (*r* = 0.36, *P* < 0.01; Pearson’s correlation analysis). **C**, **D** External FR or functional SA was correlated with the impingement distance in revision THA patients (*r* = 0.30, 0.46, respectively; *P* < 0.01; Pearson’s correlation analysis)

Among the 51 patients with 55 hips who underwent revision THA, 45 hip cases (81.8%) had external FR with a mean angle of 13.0° [standard deviation ± 12.6°] postoperatively, including 14 hip cases (25.5%) with a more than 30° external FR with a maximum of 56° in revision THA (Fig. 3A).

**Fig. 4:**
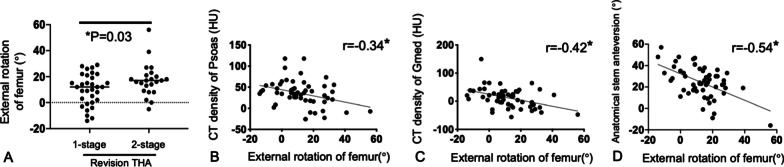
Patient and surgical factors associated with external rotation of the femur. **A** 2-stage revision total hip arthroplasty had higher external FR than 1-stage revision total hip arthroplasty (mean 17.2 ± 12.7° vs. 10.1 ± 12.0°; *P* = 0.03). **B**–**D** CT densities for the psoas and gluteus medius muscles, and anatomical stem anteversion were negatively correlated with the external rotation of the femur (*R* = 0.34, 0.42, and 0.54, respectively; *P* < 0.01; Pearson’s correlation analysis)

The external FR increased the SA from anatomical to functional (from mean 24.7° to 36.7°; *P* = 0.01). The significant correlation between the external rotation of the femur and functional SA (*r* = 0.36) indicated the importance of the FR angle on functional SA (Fig. 3B). Both external FR and the functional SA showed a significantly negative correlation with an impingement distance in the leg extension position (*r* = 0.30, 0.46, respectively; Fig. 3C and 3D). As a surgical factor, 2-stage revision THA showed a significantly higher external FR than 1-stage revision THA (mean 17.2 ± 12.7° vs. 10.1 ± 12.0°, *P* = 0.03; Fig. 4A).

Among the patient factors, the CT densities of psoas, iliac, and Gmed muscles and presence of infection were significantly associated with an increased external FR angle (Table 2; Figs. 4B and 4C). In contrast, the postoperative anatomical SA was significantly negatively correlated with external FR (Fig. 4D). In multivariate analysis, postoperative anatomical SA; CT densities of the psoas, Gmax, and Gmed muscles; and 2-stage revision were identified as independent factors associated with an external FR with the following regression equation (*R*^2^ = 0.559) (Table 3):

1 $$\begin{aligned} & {\text{Postoperative FR angle}} = 29.5 - 0.399 \times {\text{postoperative anatomical stem anteversion}} - 0.129 \\ & \quad \times {\text{CT density of psoas}} + 0.129 \times {\text{CT density of Gmax}} - 0.172 \\ & \quad \times {\text{CT density of Gmed}} - 3.291 \times {\text{revision surgery }}(1\;{\text{stage:}}\;0,2\;{\text{stage:}}\;1{)} \\ \end{aligned}$$

**Table 2:** Factors correlated with femoral rotation

Person’s correlation	*r*	*P* value
Age (years)	− 0.01	0.93
Body mass index (kg/m^2^)	0.10	0.740
Preoperative femoral rotation	0.11	0.44
Leg length extension	− 0.26	0.06
Change of femoral offset	− 0.14	0.29
Postoperative anatomical stem anteversion	− 0.54	0.001*
Size of psoas muscle	− 0.09	0.53
Size of iliac muscle	− 0.17	0.22
Size of gluteus maximus	− 0.05	0.71
Size of gluteus medius	− 0.09	0.49
CT density of psoas muscle	− 0.29	0.03*
CT density of iliac muscle	− 0.31	0.03*
CT density of gluteus maximus	− 0.17	0.22
CT density of gluteus medius	− 0.39	0.004*

**Table 3:** Multiple regression analysis for predicting femoral rotation after surgery in revision total hip arthroplasty patients

Variable	*B*	*β*	*P*-value
Constant	29.5	–	0.000
2 stage revision	− 3.291	− 0.26	0.01
Postoperative Anatomical stem anteversion	− 0.399	− 0.46	0.000
CT density of psoas muscle	− 0.129	− 0.31	0.000
CT density of gluteus maximus (Gmax)	0.129	0.26	0.03
CT density of gluteus medius (Gmed)	− 0.172	− 0.48	0.000
Regression formula for angle of postoperative femoral rotation	29.5 − 0.399 × postoperative anatomical stem anteversion − 0.129 × CT density of psoas + 0.129 × CT density of Gmax − 0.172 × CT density of Gmed (− 3.291 with 2 stage revision) (*R*^2^ = 0.559)		

Postoperative hip dislocation occurred in 8 hips during the follow-up period. Although the anatomical SA was not significantly different between revision THA patients with dislocation and non-dislocation, external FR and functional SA were significantly high in patients with dislocation (Table 4). The other factors of impingement distance, CT density of psoas, Gmed, and Gmax, body mass index, number of past operation, and ratio of 2-stage revision THA were also significantly different between patients with dislocation and non-dislocation (Table 4). Increased external FR, low impingement distance, low CT density of the psoas, Gmed muscle, BMI, 2-stage revision THA, and numbers of past operations were identified as significant risk factors for hip dislocation (Table 5).

**Table 4:** Comparison of factors between dislocation (*n* = 8) and non-dislocation (*n* = 47)

Factors	Data	*P* value
Comparison of ratio for dislocation (%)		Chi-square test
Diagnosis	PJI: 5/23(22%) versus aseptic: 3/32(9%)	< 0.01
Surgical procedure	2-stage: 5/22(23%) versus 1-stage: 3/33(9%)	< 0.01
Comparison of mean values [standard deviation]		*t* test
BMI (kg/cm^2^)	27.2 [1.5] versus 23.2 [0.44]	< 0.01
CT density of Psoas muscle (HU)	13.3 [25.1] versus 37.4 [23.2]	< 0.01
CT density of gluteus maximus (HU)	− 6.9 [25.1] versus 13.5 [22.1]	< 0.05
CT density of gluteus medius (HU)	− 6.9 [13.6] versus 24.5 [28.6]	< 0.01
External rotation of femur (°)	18.5 [9.1] versus 4.8 [14.3]	< 0.01
Functional stem anteversion (°)	43.6 [10.5] versus 34.5 [12.5]	< 0.05
Impingement distance(mm)	13.7 [9.7] versus 21.3 [9.1]	< 0.05
Numbers of past operation	2.4 [1.8] versus 1.5 [0.9]	< 0.05

**Table 5:** Odds ratio for postoperative dislocation

	Odd ratio	95% confidence interval		*P* value
		Lower limit	Upper limit	
External rotation of femur	1.061	1.011	1.114	0.016
Impingement distance	0.901	0.820	0.991	0.031
CT density of psoas muscle	0.957	0.926	0.988	0.007
CT density of gluteus medius	0.963	0.938	0.989	0.005
BMI	1.178	1.022	1.358	0.024
2-stage revision THA	5.268	1.620	17.131	0.006
Numbers of past operation	2.289	1.322	3.963	0.003

## Discussion

Operative planning to achieve an optimal combined cup and SA is important for successful THA [1–3]. Previous reports revealed that internal changes in the FR occur in primary THA patients [7, 19]. As an excessive external femoral position is occasionally observed at an early stage after surgery in revision THA cases, we first focused on the FR angle and functional SA, as well as their relation to the posterior impingement distance in revision THA patients. In our study population, more than 80% of revision THA patients showed external FR that functionally increased the SA and posterior impingement risk in leg extension. Because the tendency of internal femoral changes in primary THA patients does not typically contribute to posterior impingement and the risk of hip dislocation, we investigated whether the external FR and impingement risk was a one cause of the higher dislocation rate in revision THA patients [8, 9, 20]. As a result, a higher external FR and lower impingement distance were identified as significant risk factor for postoperative hip dislocation in our cohort, indicating that revision THA patients with an excessive external FR position require careful assessment of hip dislocation.

Abductor deficiency contributes to hip instability [9, 10, 13–15, 20], indicating the necessity of quantifying the size and density of muscles around the hip joint and assessing the associations between them and the femoral rotational angle or hip dislocation. For this purpose, we utilized previously established methods to evaluate the muscle size and density via cross-sectional CT imaging, which can reflect muscle strength [16]. Interestingly, our CT analysis revealed a significant correlation between muscle weakness in the psoas, iliac, or Gmed muscles and external FR. This can be explained by the internal rotational function of these muscles, in contrast to the Gmax function during external rotation. Therefore, the greater increase in FR in patients with septic loosening who mostly underwent 2-stage revision THA likely occurred because of the atrophic effect of the greater surgical intervention or longer disuse period on these muscles in 2-stage revision patients. Our CT analysis also revealed a negative correlation between the postoperative SA and FR, similar to the findings of a previous study of primary THA patients [7]. This paradoxical effect may also be explained by the fact that a higher SA induces higher tensioning of the iliopsoas or Gmed but lower tensioning of the Gmax [7].

Multiple regression analysis revealed the independent functions of the psoas and Gmed muscles on the internal FR. Nevertheless, the psoas and Gmed muscles reportedly contribute to hip stability [9, 10, 13–15]. The significant correlations of these muscles with FR indicate that the excessive external FR position in revision THA patients is an important clinical sign of muscle weakness in the psoas and Gmed muscles. As these muscles are well-known and important stabilizers of the hip joint [9, 10, 13–15], we additionally investigated the association of these muscles with the hip dislocation risk. As a result, low CT densities of the psoas and medius muscle were also identified as risk factors for hip dislocation. Therefore, patients with an excessive external FR may have the risk factors of hip instability because of muscle atrophy and hip impingement due to an increased functional SA for postoperative hip dislocation.

Our study had several limitations. First, more than 50% of the revision THA patients treated at our hospital were not included in retrospective evaluations because of a lack of medical records, clinical follow-up, and image assessment. However, we included all dislocation cases during the study period that may have resulted in successful analysis of the association of FR with dislocation. Secondly, we did not evaluate mid- and long-term changes in FR in our study cases. However, our study is the first to demonstrate the tendency towards an external FR, and to reveal an association between the lower muscle CT density and early postoperative posture in a revision THA cohort. SA modification or muscle strengthening may be required in these patients; however, further studies are needed to validate this possibility.

In conclusion, more than 80% of revision THA caused an external FR that functionally increased the SA and impingement risk in leg extension, particularly in hips with 2-stage revision with psoas and Gmed muscle atrophy. Revision THA patients with an excessive external FR may require careful monitoring for hip dislocation due to instability and impingement.

## Data Availability

The datasets used and/or analyzed during the current study are available from the corresponding author on reasonable request.

## Abbreviations

SA: Stem anteversion
THA: Total hip arthroplasty
FR: Femoral rotation
Gmax: Gluteus
Gmed: Medius
BMI: Body mass index
